# Effectiveness of the DigniCap® Scalp Cooling System for the Prevention of Chemotherapy-Induced Alopecia: An Observational Study in a Uruguayan Cohort

**DOI:** 10.7759/cureus.114133

**Published:** 2026-08-07

**Authors:** Dahiana Amarillo, Natalia Camejo, Cecilia Castillo, Maria Planinich, Melanie Garcia, Belen Insagaray, Joaquin Manzanares, Belen Acevedo, Sofia Benavides, Juliana Manzino, Paola Muiño, Guillermina Servetto, Maria Guerrina, Virginia Suaya, Catherine Mate, Gabriela Latorraca, Gabriel Krygier

**Affiliations:** 1 Department of Clinical Oncology, Hospital de Clínicas, School of Medicine, University of the Republic, Montevideo, URY; 2 Department of Clinical Oncology, School of Medicine, University of the Republic, Montevideo, URY; 3 Department of Medical Oncology, School of Medicine, University of the Republic, Montevideo, URY

**Keywords:** antineoplastic agents, breast neoplasms, drug-induced alopecia, quality of life, scalp cooling

## Abstract

Background: Chemotherapy-induced alopecia is one of the most frequent and distressing adverse effects of systemic treatment in patients with breast cancer, with a relevant negative impact on body image and quality of life. Scalp cooling represents the most promising available preventive strategy; however, evidence from middle-income countries and real-world clinical practice remains limited. The objective of this study was to evaluate the effectiveness and tolerability of the DigniCap® scalp cooling system (Dignitana AB, Lund, Sweden) for preventing chemotherapy-induced alopecia (CIA), as well as its impact on quality of life, among Uruguayan patients with early breast cancer (EBC) receiving adjuvant or neoadjuvant treatment.

Methods: An observational, analytical, longitudinal, ambispective, single-arm study was conducted, including patients with early breast cancer who used the DigniCap® system between April 2022 and August 2025. The degree of alopecia was assessed using the Dean scale, with success defined as hair loss ≤50%. Quality of life, symptom perception, and psychosocial impact were evaluated using validated questionnaires and scales, including the European Organisation for Research and Treatment of Cancer Breast Cancer-Specific Quality of Life Questionnaire (EORTC QLQ-BR23).

Results: A total of 137 patients were included, with a median age of 48 years. Treatment with DigniCap® was completed by 89.1% of patients. Alopecia assessment using the Dean scale was feasible in 95.6% of the cohort (n=131); of these evaluable patients, 86.3% (n=113) achieved alopecia ≤50% (treatment success). No statistically significant differences were observed according to age, institution of care, or chemotherapy regimen. The most frequent adverse events were headache, pruritus, and local discomfort, generally mild to moderate in intensity and well tolerated. A positive perception of body image and a favorable impact on quality of life were observed.

Conclusions: The DigniCap® scalp cooling system demonstrated high effectiveness and good tolerability in preventing chemotherapy-induced alopecia in this cohort. The observed success rate was within the upper range reported in international series, although cross-study comparisons should be interpreted cautiously given differences in patient populations, chemotherapy regimens, and study methodology. These findings support the implementation of DigniCap® as a safe and well-tolerated strategy for the prevention of chemotherapy-induced alopecia, with a potential favorable impact on patients' body image during oncologic treatment.

## Introduction

Breast cancer is the most frequent malignant tumor and the leading cause of cancer death in women, both worldwide and in Uruguay [1,2]. In early stages, adjuvant and neoadjuvant chemotherapy has demonstrated a significant benefit in overall survival and disease-free survival; however, its use is associated with adverse effects that have a relevant impact on patients' quality of life [3].

Among these effects, chemotherapy-induced alopecia (CIA), defined as partial or total hair loss in areas of normal growth, is one of the most visible and emotionally distressing, particularly in breast cancer patients. Its prevalence varies according to the chemotherapy regimen, affecting approximately 65% of patients treated with conventional chemotherapy and up to 89% of those receiving taxane-based regimens [4,5]. Hair loss usually begins one to three weeks after the start of treatment and, although classically considered reversible, up to 40% of patients may develop persistent or permanent alopecia, defined as absent or incomplete hair recovery six months or more after chemotherapy completion, a phenomenon most frequently associated with taxane use [6,7].

The psychosocial impact of CIA is considerable. Between 47% and 58% of patients identify hair loss as the most traumatic aspect of cancer treatment, and up to 8% might even decline chemotherapy for this reason [4,5]. Alopecia negatively affects body image, self-esteem, sexuality, and quality of life, and may promote social isolation and reduce treatment adherence [8,9].

Since no effective curative treatments exist for persistent chemotherapy-induced alopecia, prevention plays a central role [4]. In this context, scalp cooling has been shown to be the most effective intervention for reducing the incidence and severity of CIA, with hair preservation rates ranging from 50% to 80%, depending on the device used and the chemotherapy regimen administered [7-10]. Its mechanism of action is based on local hypothermia, which induces vasoconstriction and reduces follicular metabolic activity, limiting the delivery and uptake of cytotoxic agents in the hair bulb [7]. Furthermore, this strategy has been shown to reduce alopecia-related distress and improve quality of life during cancer treatment [10].

Automated cooling devices, such as DigniCap® (Dignitana AB, Lund, Sweden), allow precise and sustained temperature control and have been widely incorporated into clinical practice. The efficacy of scalp cooling varies according to the chemotherapy regimen, with better results observed in taxane-based regimens and lower hair preservation rates in anthracycline-containing combinations [11-13]. Meta-analytic data show an overall effectiveness of approximately 60% for hair preservation [11] and a significant reduction in persistent alopecia six months after treatment completion [10]. In particular, a meta-analysis including over 654 patients (66% with breast cancer) demonstrated that scalp cooling significantly reduces the risk of alopecia greater than 50% (RR 0.57; 95% CI 0.45-0.72; p<0.00001) compared to controls [11]. Furthermore, the SCALP trial showed that alopecia ≤50% was achieved in 66.3% of the DigniCap® group versus 0% in controls (p<0.001), with simultaneous improvement in body image perception and self-esteem [8]. In terms of safety, scalp cooling has a favorable profile, with generally mild to moderate adverse events and no evidence of increased risk of scalp metastases or compromise of oncologic outcomes [11,14]. Based on this evidence, international guidelines from the National Comprehensive Cancer Network (NCCN) and European Society for Medical Oncology (ESMO) recommend its use for the prevention of CIA in breast cancer patients, with a level of evidence IIA [15,16].

However, most of the available evidence comes from studies conducted outside Latin America, and extrapolation of these results to other populations should be interpreted with caution [12,17-19]. No published studies have evaluated the efficacy of scalp cooling in Uruguay; however, a preliminary study conducted at Hospital de Clínicas in 40 patients with early breast cancer (EBC) showed hair loss below 50% in 67.5% of cases, with good device tolerability [20]. The present study aims to expand this experience and evaluate the effectiveness of the DigniCap® system in a larger Uruguayan cohort. The primary objective was to evaluate the effectiveness of the DigniCap® scalp cooling system, defined as hair preservation with Dean scale alopecia ≤50%, for the prevention of CIA in Uruguayan patients with early breast cancer receiving adjuvant or neoadjuvant chemotherapy. Secondary objectives were to compare the success rate according to age group, type of healthcare institution (public versus private sector), and chemotherapy regimen received; to describe the safety and tolerability of the system by evaluating associated adverse events and the percentage of patients who completed treatment; to analyze patients' subjective perception of device effectiveness and the consequences of alopecia; and to compare quality of life, as measured by the European Organisation for Research and Treatment of Cancer Breast Cancer-Specific Quality of Life (EORTC QLQ-BR23) questionnaire, according to treatment outcome.

## Materials and methods

An observational, analytical, longitudinal, ambispective, single-arm study was conducted. Uruguayan patients with a diagnosis of early breast cancer (EBC) who received adjuvant or neoadjuvant chemotherapy between April 2022 and August 2025 and used the DigniCap® scalp cooling system (Dignitana AB, Lund, Sweden; DigniCap® Delta model) during treatment were included. Consecutive non-probability sampling was used, including all patients who met the eligibility criteria during the study period. Given the observational design and the absence of a comparison group, no formal a priori sample size calculation was performed. As this was a retrospective/ambispective, single-arm observational study without a comparator group, it was not registered in a clinical trial registry.

Inclusion criteria were: age ≥18 years; diagnosis of EBC with indication for adjuvant or neoadjuvant chemotherapy; use of the DigniCap® system during treatment; patients from the Hospital de Clínicas and public health services under the Administration of State Health Services (ASSE), defined as "Internal," or users of the private health system, defined as "External"; and written informed consent for the use of DigniCap®, completion of questionnaires, and photographic documentation. Exclusion criteria included: concurrent malignancy or prior receipt of alopecia-inducing chemotherapy within the previous two years; autoimmune disease causing alopecia; whole-brain radiotherapy; or cold agglutinin disease.

The DigniCap® system was applied starting 30 minutes before the onset of chemotherapy infusion and remained in place throughout the entire infusion period. Scalp temperature was maintained at approximately +3°C. Cooling was continued for 120 minutes after completion of the infusion, uniformly across all chemotherapy regimens included in the study.

Data collection was performed by oncologists from the Clinical Oncology Service of the Hospital de Clínicas. Before the first chemotherapy cycle, a standardized photographic record of the scalp was obtained using five photographs, which was repeated at each visit prior to subsequent cycles and three weeks after treatment completion.

At each cycle, patients completed the EORTC QLQ-BR23 quality-of-life questionnaire [21], used with permission from the EORTC Quality of Life Group, as well as a multiple-choice questionnaire assessing perceived symptoms associated with DigniCap® use (Appendix 1) and another evaluating self-perception of alopecia and its consequences (Appendix 2). All questionnaires were self-administered by patients, either at home or during the clinical visit, according to their preference; patients were able to seek clarification from the treating physician or a member of the research team when needed. Questionnaire completion was voluntary; not all patients responded to every item in each instrument. For each item, the number of valid responses available is reported (N per item). Subsequent clinical follow-up was conducted as part of routine care at each patient's respective healthcare provider.

The degree of alopecia was assessed using the Dean scale [22]. At least three investigators independently evaluated the final photographs compared to the baseline photographs, blinded to each patient's chemotherapy regimen and institutional origin (internal versus external), assigning a single score per patient by consensus. Treatment success was defined as alopecia ≤50% (grades 0, 1, and 2) and treatment failure as alopecia >50% (grades 3 and 4). Patients who discontinued DigniCap® use before completing chemotherapy were retained in the descriptive and safety/tolerability analyses; however, those without an evaluable final photographic record were excluded from the Dean-scale effectiveness analysis.

Statistical analysis

Each patient's responses throughout treatment were compiled into a single database. For the symptom perception and alopecia self-perception questionnaires, the worst value reported by each patient for each item was selected in order to evaluate the maximum impact experienced. The EORTC QLQ-BR23 questionnaire was analyzed according to standard recommendations, grouping items by functional or symptomatic domain, averaging the corresponding values, and transforming them into scales. In functional scales, higher scores indicated better functioning, while in symptom scales, they represented greater symptomatology. Quantitative variables were described using medians and ranges. Fisher's or chi-square tests were used to evaluate associations between qualitative variables. The normality of continuous variables was assessed using the Shapiro-Wilk test, and comparisons between independent groups were performed using the Mann-Whitney U test. A p-value <0.05 was considered statistically significant. Statistical analysis was performed using R software version 4.4.1 (R Foundation for Statistical Computing, Vienna, Austria).

Ethical considerations

This study was conducted in accordance with international ethical standards for biomedical research, the Declaration of Helsinki, MERCOSUR regulations on clinical trial standards, and the national research regulations approved by the National Ethics Commission in 2019. National legislation on personal data protection (Law No. 18,331) was also respected. All participants provided written informed consent prior to inclusion in the study. Confidentiality, privacy, and anonymity of patients and their personal data were guaranteed. Photographic material was edited to prevent participant identification, ensuring protection of their identity. The study protocol was reviewed and approved by the Research Ethics Committee of Hospital de Clínicas “Dr. Manuel Quintela,” Universidad de la República, under reference number 16-25E, dated June 25, 2025.

## Results

A total of 137 patients with early breast cancer treated with the DigniCap® scalp cooling system were included. The clinico-demographic characteristics of the cohort are summarized in Table 1. The median age was 48 years (range: 26-79), and 62.0% of patients came from the private health system.

**Table 1:** Clinico-demographic characteristics of the study cohort (N=137). Data are expressed as n (%) for categorical variables. Age is expressed as median (range). No statistical comparisons were performed for this descriptive table. -: not applicable (category header row). For the "Median age (range)" row, the n column shows the median value and the % column shows the range.

Variable	n	%
Age	-	-
Median age (range)	48	26-79
Age <50 years	78	56.9
Age ≥50 years	59	43.1
DigniCap® treatment status	-	-
Complete	122	89.1
Incomplete	15	10.9
Healthcare institution	-	-
Public health system (internal)	52	38.0
Private health system (external)	85	62.0
Chemotherapy regimen	-	-
Carboplatin, docetaxel, trastuzumab, and pertuzumab	29	21.2
Doxorubicin and cyclophosphamide followed by taxanes	34	24.8
Doxorubicin, cyclophosphamide, carboplatin, and paclitaxel (generally with pembrolizumab)	17	12.4
Docetaxel and cyclophosphamide	41	29.9
Dose-dense doxorubicin and cyclophosphamide followed by taxanes	16	11.7

Use and adherence to the DigniCap® system

A total of 89.1% (n=122) of patients completed the full course of treatment with DigniCap®. Reasons for discontinuation were related to device tolerability or associated adverse events: 40.0% (n=6) discontinued due to cold intolerance, 40.0% (n=6) due to perceived alopecia, 6.7% (n=1) due to headache, and 13.3% (n=2) did not specify a reason. These patients were included in the descriptive analysis; however, in six cases, the degree of alopecia could not be evaluated using the Dean scale. Regarding the chemotherapy regimens administered, both anthracycline-containing and anthracycline-free regimens were used, with anthracycline-free regimens being the most frequent in the cohort (Table 1).

Efficacy according to the Dean scale

**Figure 1:**
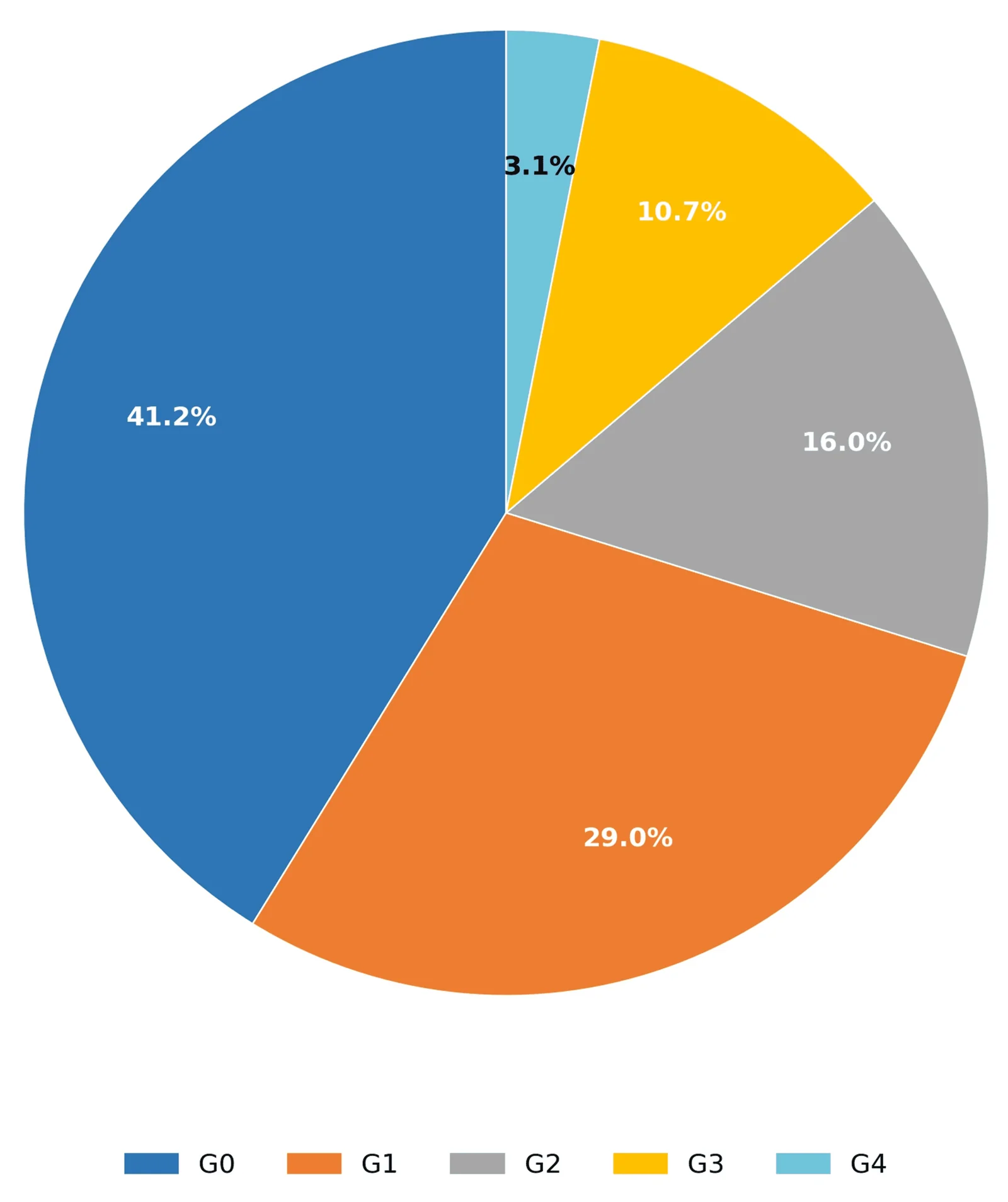
Distribution of alopecia grades observed according to the Dean scale. Distribution of alopecia grades observed according to the Dean scale (N=131). G0: no hair loss; G1: >0-25%; G2: >25-50%; G3: >50-75%; G4: >75% hair loss. Figure created using R version 4.4.1 with the ggplot2 package (R Foundation for Statistical Computing, Vienna, Austria).

Evaluation of alopecia using the Dean scale was possible in 95.6% (n=131) of patients. A success rate (hair loss ≤50%) was observed in 86.3% (n=113). The distribution of alopecia grades is presented in Figure 1. No statistically significant association was observed between the efficacy of the DigniCap® system and age at treatment, grouped as <50 and ≥50 years (p=0.267). Likewise, no significant differences in success rate were identified between patients from the public and private health systems (p=0.459). Regarding the chemotherapy regimens administered (Table 1), no statistically significant association was found between the type of regimen and scalp cooling efficacy (p=0.449). Similarly, when comparing regimens with and without anthracyclines, no significant differences were observed in outcome according to the Dean scale (p=0.234).

Adverse events and tolerability

**Figure 2:**
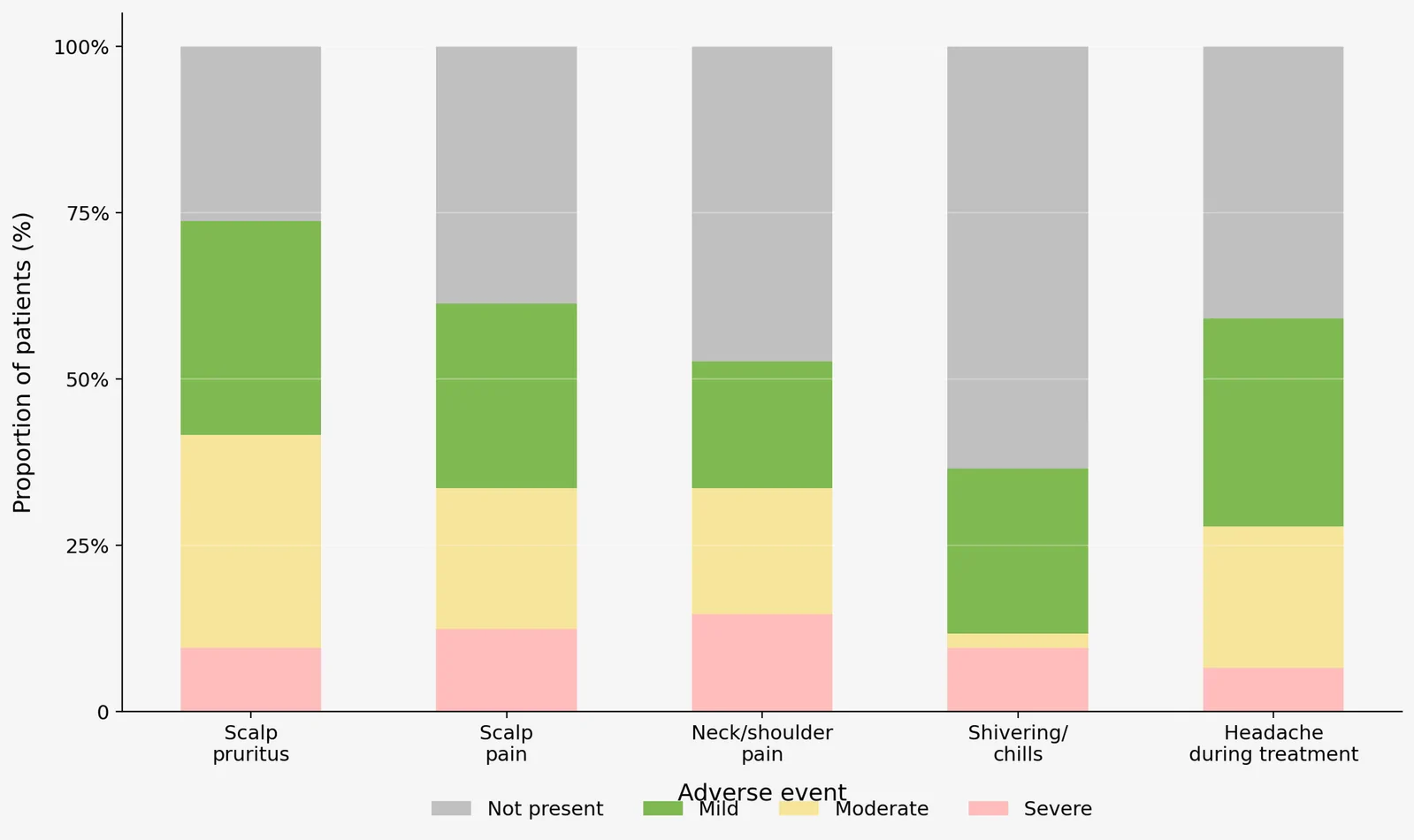
Proportion of patients reporting each adverse event associated with DigniCap® use, stratified by intensity. X-axis: type of adverse event; Y-axis: proportion of patients (%). Colors indicate severity: not present (grey), mild (green), moderate (yellow), severe (pink). Figure created using R version 4.4.1 with the ggplot2 package (R Foundation for Statistical Computing, Vienna, Austria).

**Figure 3:**
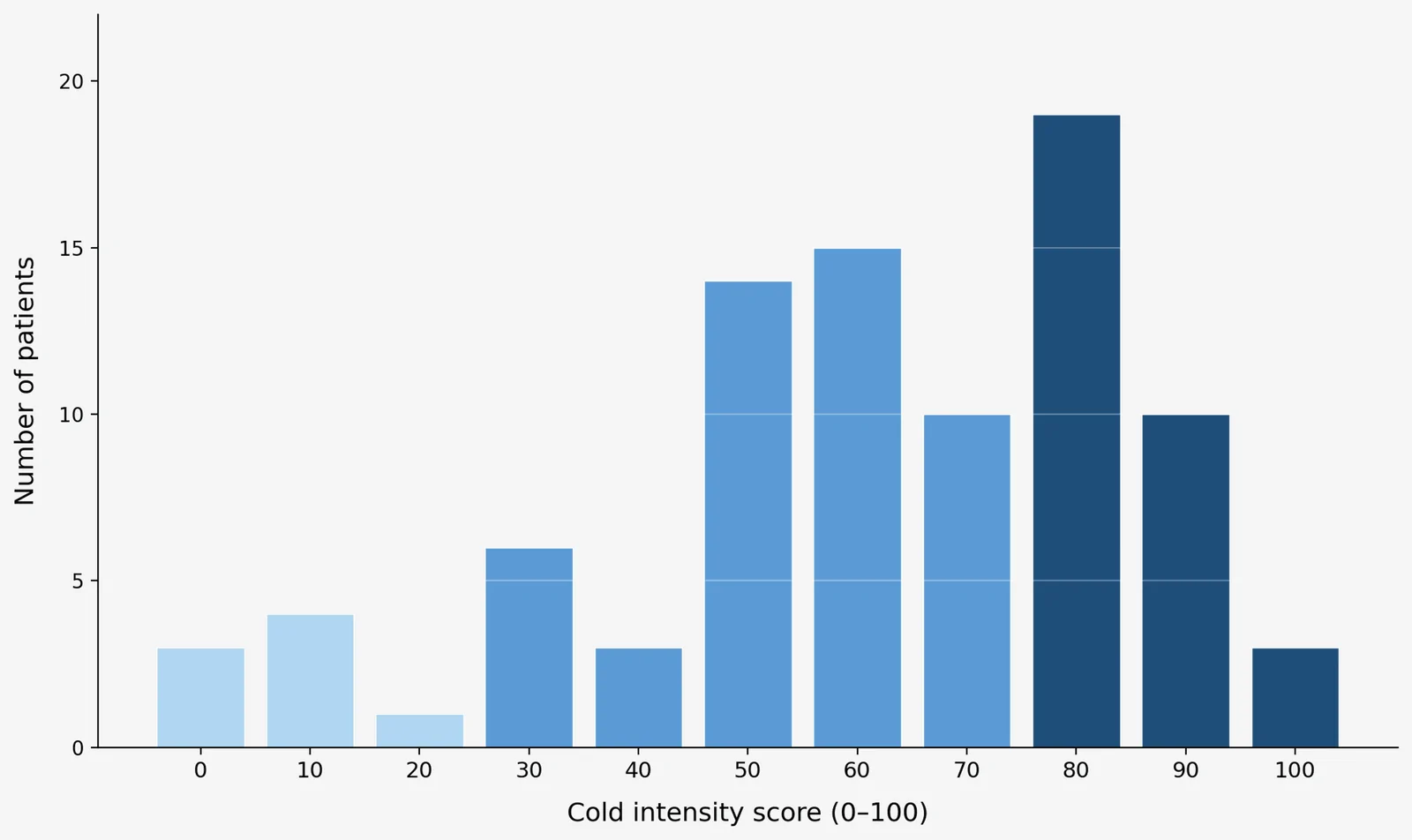
Cold perception during DigniCap® use among patients who completed the cold perception scale (N=88). X-axis: cold intensity score (0-100); Y-axis: number of patients. Figure created using R version 4.4.1 with the ggplot2 package (R Foundation for Statistical Computing, Vienna, Austria).

The most frequently reported adverse event was headache, reported by 78% (n=107) of patients, mostly occasionally. During DigniCap® use, headache occurred in 59.1% (n=81), and only 13.9% (n=19) required analgesia for its management. Other frequent adverse events included scalp pruritus (73.7%; n=101), scalp pain (61.3%; n=84), neck and/or shoulder pain (52.6%; n=72), and tremors or chills (36.5%; n=50) (Figure 2). Less frequent adverse events of gastrointestinal origin were also reported, mainly nausea (n=5), vomiting (n=1), and abdominal pain (n=1). At the systemic level, dizziness (n=4), sweating (n=2), drowsiness (n=1), and skin rash (n=1) were recorded. Regarding cold perception during DigniCap® use, 80.7% of patients (n=71 out of 88 who completed the cold perception scale) reported an intensity equal to or greater than 50 points on the scale used (Figure 3). The vast majority reported adequate understanding of the instructions (72.5%; n=99) and no relevant difficulties in adjusting the cap (92%; n=126).

Self-perception of alopecia

Analysis of body self-perception showed that most patients did not feel inhibited by their appearance or perceive themselves as less physically or sexually attractive, and no social avoidance attributable to alopecia was observed. However, both qualitative and quantitative perception of hair loss were significantly associated with outcome according to the Dean scale (p=0.006 and p<0.001, respectively). Detailed results are presented in Tables 2, 3.

**Table 2:** Alopecia self-perception results according to treatment outcome (N=81*). *N=81: patients who completed the alopecia self-perception questionnaire (Appendix 2), representing a subset of the 131 patients with an evaluable Dean scale assessment. Success: alopecia ≤50% (grades 0-2); Failure: alopecia >50% (grades 3-4). †N=80 for this item due to one missing response. p-values calculated using Fisher's exact test or chi-square test as appropriate. Bold p-value indicates statistical significance (p<0.05).

Variable	Not at all/a little success	Not at all/a little Failure	Quite a bit/very much success	Quite a bit/very much Failure	p-value
Self-conscious about appearance	62 (76.5%)	5 (6.2%)	12 (14.8%)	2 (2.5%)	0.409
Less physically attractive	58 (71.6%)	5 (6.2%)	16 (19.8%)	2 (2.5%)	0.672
Dissatisfied with appearance when dressed	63 (77.8%)	4 (4.9%)	11 (13.6%)	3 (3.7%)	0.061
Difficulty looking at body when undressed	62 (76.5%)	5 (6.2%)	12 (14.8%)	2 (2.5%)	0.409
Less sexually attractive	57 (70.4%)	5 (6.2%)	17 (21.0%)	2 (2.5%)	0.738
Avoids seeing people†	67 (83.8%)	6 (7.5%)	6 (7.5%)	1 (1.3%)	0.587
Qualitative perception of hair loss	40 (49.4%)	0 (0.0%)	34 (42.0%)	7 (8.6%)	0.006

**Table 3:** Quantitative perception of hair loss according to treatment outcome (N=80*). Percentages calculated within each outcome group (Success n=73; Failure n=7). p-values calculated using Fisher's exact test. Success: alopecia ≤50% (grades 0-2); Failure: alopecia >50% (grades 3-4). *One patient from the 81 who completed the self-perception questionnaire did not respond to this item.

Hair loss percentage	Success n (%)	Failure n (%)	p-value
>0 to ≤25%	36 (49.3%)	0 (0.0%)	<0.001
>25 to ≤50%	18 (24.7%)	1 (14.3%)	<0.001
>50 to ≤75%	13 (17.8%)	2 (28.6%)	<0.001
>75%	6 (8.2%)	4 (57.1%)	<0.001

Quality of life

Analysis of the EORTC QLQ-BR23 questionnaire showed globally favorable scores in the dimensions of body image, sexual function and satisfaction, and future health concerns. When comparing patients classified as “Success” and “Failure” according to the Dean scale, no statistically significant differences were observed in any of the dimensions evaluated (p>0.05 for all comparisons). Results are summarized in Table 4.

**Table 4:** EORTC QLQ-BR23 quality of life results by domain (N=131). Mean scores ± SD represent the full evaluable cohort. U and p-values are from the Mann-Whitney U test comparing Success vs. Failure groups (Dean scale). Scores range from 0 to 100; for functional scales, higher scores indicate better functioning; for symptom scales, higher scores indicate greater symptom burden. No statistically significant differences were observed in any domain (all p>0.05). SD: standard deviation; EORTC QLQ-BR23: European Organisation for Research and Treatment of Cancer Breast Cancer-Specific Quality of Life Questionnaire.

Dimension	Systemic therapy side effects	Body image	Future health concerns	Sexual functioning	Sexual enjoyment	Arm/shoulder symptoms	Breast symptoms
Mean score ± SD	39.8 ± 18.5	86.7 ± 22.7	56.9 ± 32.5	81.6 ± 21.0	63.6 ± 34.7	24.5 ± 19.3	24.9 ± 15.5
U (Mann-Whitney)	228	278	340	201	88	327	330
p-value	0.538	0.830	0.204	0.259	0.139	0.237	0.222

## Discussion

In our study, 86.3% of patients achieved a degree of alopecia ≤50%, confirming the high efficacy of scalp cooling with DigniCap®. This finding is consistent with international evidence, which positions scalp cooling as the most effective strategy for preventing chemotherapy-induced alopecia in breast cancer, with overall hair preservation rates close to 60% and better results in taxane-based regimens [1,7,14,17]. In particular, our success rate of 86.3% is comparable to that reported in the Korean randomized trial by Kang et al. (86.5%) [10], and higher than rates observed in prospective studies conducted in Italy (60-68%) [12,17], Germany (39.3%) [18], the United States (66.3%) [8], and Singapore [23]. This variability across studies likely reflects differences in chemotherapy regimens, patient populations, and study methodologies, rather than a true superiority of our results; in particular, studies with a higher proportion of taxane-based regimens without anthracyclines consistently report higher success rates. Our results are also consistent with the Uruguayan experience from the Digni-T-Uy trial [20] and with the limited available Latin American evidence, including a study conducted in Mexico that demonstrated high effectiveness of the method and the influence of chemotherapy regimen and sequence [13]. To date, no published studies have formally evaluated the efficacy of scalp cooling in Uruguay; therefore, our results provide original regional evidence and represent, to our knowledge, the first published experience in Uruguayan patients, reinforcing the applicability of international data in this context.

The high efficacy observed in our cohort may be explained, in part, by the type and sequence of chemotherapy regimens used. The international literature consistently demonstrates that scalp cooling with DigniCap® is more effective in taxane-based regimens, with hair preservation rates frequently exceeding 80%, while efficacy decreases in regimens including anthracyclines, especially when these are administered before taxanes [8,12,13]. It has also been shown that initiating treatment with taxanes is associated with better outcomes compared with the reverse sequence [12,13]. The lack of statistically significant differences between regimens in our study may be attributed to the limited sample size, which reduces statistical power to detect differences between subgroups. Notably, all six patients who discontinued treatment due to perceived alopecia had received exclusively anthracycline-based regimens (AC-CP, AC-T, or AC-T-DD), of whom five were classified as Failure according to the Dean scale. This finding suggests that discontinuation due to alopecia was linked to the absence of clinical benefit in patients receiving regimens with higher hair toxicity, rather than to device intolerance.

Consistent with previous studies, age did not appear to have a relevant impact on the efficacy of scalp cooling in our population [4,5]. On the other hand, while access to scalp cooling may vary by healthcare setting, the evidence suggests that, when used correctly, its efficacy is comparable between public and private systems, provided that placement and cooling protocols are properly followed [8,24,25].

Discontinuation of the DigniCap® system was low (10.9%) and was mainly due to cold intolerance and perceived alopecia, consistent with the literature. Previous studies identify progressive alopecia and procedure intolerance, generally due to mild adverse events, such as headache or intense cold, as the main reasons for discontinuation [12,17,26]. These findings reinforce that scalp cooling is a globally well-tolerated strategy, and that discontinuation is usually linked to perceived benefit rather than serious adverse events [8,12,26].

Adverse events associated with DigniCap® use were frequent but mostly mild to moderate, with headache, pruritus, scalp pain, and the sensation of cold being most prominent. The discontinuation rate was low and was mainly attributable to cold intolerance or perceived alopecia. These findings are consistent with the international literature, which describes scalp cooling as a well-tolerated procedure with transient adverse events and a low incidence of serious complications [11,17,26]. Several strategies may help minimize these adverse events and improve tolerability. A recent systematic review and meta-analysis concluded that clinical comfort strategies, including supportive medications (e.g., prophylactic analgesia for headache) and reinforced patient education before and during treatment, could meaningfully improve scalp cooling tolerability and support its broader implementation [26]. In addition, a randomized pilot study found that the use of electric hand warmers during scalp cooling sessions significantly improved thermal comfort compared with observation alone, without compromising cooling efficacy at the scalp, suggesting that simple adjunctive warming measures for the extremities may help reduce cold-related discomfort and, potentially, discontinuation rates [27].

In our cohort, DigniCap® use was not associated with statistically significant differences in quality of life dimensions as evaluated by the EORTC QLQ-BR23 between patients classified as “Success” and “Failure” (p>0.05). Given that the analysis considered the worst value recorded throughout treatment, it was expected that these differences would not reach statistical significance. These results are consistent with international evidence, which shows that alopecia reduction does not always translate into significant improvements in overall quality of life [8,26]. Nevertheless, a trend toward better body image perception was observed in patients with “Success,” likely associated with hair preservation, reinforcing the psychosocial benefit of scalp cooling in a context of high emotional vulnerability [19,24,26]. Furthermore, the significant correlation between qualitative and quantitative self-perceptions of hair loss and Dean scale outcomes (p=0.006 and p<0.001, respectively) supports the convergent validity of this instrument for assessing scalp cooling efficacy.

The main strengths of this study include the inclusion of a large cohort of Uruguayan patients treated under real-world clinical practice conditions, the use of validated instruments for the evaluation of alopecia and quality of life, and the comprehensive analysis of efficacy, tolerability, and adverse events of the DigniCap® system. It also constitutes the first published experience on scalp cooling in Uruguay. The main limitations include the single-arm design and the absence of a control group, which preclude direct comparisons with patients not receiving scalp cooling, as well as the heterogeneity of chemotherapy regimens and healthcare institutions, which may have limited the detection of differences between subgroups. Additionally, as an observational study without random allocation to scalp cooling, selection bias cannot be excluded, since patients who elected or had access to DigniCap® may differ systematically from the broader population of patients receiving chemotherapy; furthermore, the observational design did not allow adjustment for potential confounding factors that may have influenced the outcomes observed. Finally, the quality of life analysis based on the worst value recorded during treatment may have attenuated differences that might have been more evident with longitudinal follow-up. Future studies should incorporate controlled designs with a comparison group, larger sample sizes, long-term follow-up to assess post-treatment hair recovery, and stratified analyses by molecular subtype and chemotherapy regimen, in order to confirm and expand upon the findings of the present study.

## Conclusions

In this Uruguayan cohort of patients with early breast cancer, the DigniCap® scalp cooling system demonstrated high effectiveness for the prevention of chemotherapy-induced alopecia, with a success rate of 86.3%, as well as good tolerability and a favorable safety profile. The majority of patients completed treatment, and adverse events were predominantly mild and transient. Although no significant differences were observed in overall quality of life scales, a positive perception of body image was evidenced, particularly among patients with hair preservation. These results support the implementation of scalp cooling as a safe and effective strategy in real-world clinical practice, providing original evidence from Uruguay and reinforcing its value as a supportive care measure during cancer treatment.

## Appendices

Appendix 1

Symptom Perception Questionnaire to be completed by the patient after each chemotherapy cycle received using DigniCap®.

- Patient number:

- Date:

- Chemotherapy cycle:

1) Over the past 20 days, what was the severity of scalp itching at its worst?

1. I did not experience it

2. Mild

3. Moderate

4. Severe

5. Very severe

2) Over the past 20 days, how often did you have headaches?

1. Never

2. Rarely

3. Occasionally

4. Frequently

5. Almost constantly

3) Over the past 20 days, what was the severity of your headache?

1. I did not experience it

2. Mild

3. Moderate

4. Severe

5. Very severe

4) Over the past 20 days, how much did your headache interfere with your daily activities?

1. Not at all

2. A little

3. Somewhat

4. Quite a bit

5. Very much

5) Over the past 7 days, how often did you experience tremors or chills?

1. Never

2. Rarely

3. Occasionally

4. Frequently

5. Almost constantly

6) Over the past 7 days, what was the severity of the tremors or chills?

1. I did not experience it

2. Mild

3. Moderate

4. Severe

5. Very severe

7) Difficulty fitting the cap during today’s treatment - what was the severity of this at its worst?

1. I did not experience it

2. Mild

3. Moderate

4. Severe

5. Very severe

8) Difficulty with pre-treatment instructions. What was the severity?

1. None

2. Mild

3. Moderate

4. Severe

5. Very severe

9) Neck/shoulder pain. What was the severity of this symptom?

1. I did not experience it

2. Mild

3. Moderate

4. Severe

5. Very severe

10) Scalp pain. What was the severity of this symptom?

1. I did not experience it

2. Mild

3. Moderate

4. Severe

5. Very severe

11) Headache during treatment. What was the severity of this symptom?

1. I did not experience it

2. Mild

3. Moderate

4. Severe

5. Very severe

12) Did you need to request analgesics during treatment?

Yes

No

13) Mark on the following scale how cold you felt today during treatment (0 = no cold at all, 100 = maximum cold).

0------10------20------30------40------50------60------70------80------90------100

Other symptoms

Do you have any other symptoms you would like to report?

Yes

No

Please specify:

Appendix 2

Questionnaire to assess self-perception of alopecia and its consequences for the patient.

1) Have you felt self-conscious about your physical appearance?

1. Not at all

2. A little

3. Quite a bit

4. Very much

2) Have you felt less physically attractive as a result of your illness or treatment?

1. Not at all

2. A little

3. Quite a bit

4. Very much

3) Have you been dissatisfied with your appearance when getting dressed?

1. Not at all

2. A little

3. Quite a bit

4. Very much

4) Have you found it difficult to look at yourself undressed?

1. Not at all

2. A little

3. Quite a bit

4. Very much

5) Have you felt less sexually attractive as a result of your illness or treatment?

1. Not at all

2. A little

3. Quite a bit

4. Very much

6) Have you avoided seeing people because of the way you felt about your appearance?

1. Not at all

2. A little

3. Quite a bit

4. Very much

7) Do you feel that you have lost hair?

1. Not at all

2. A little

3. Quite a bit

4. Very much

8) What percentage of hair loss do you think you have experienced?

1. 0 to ≤25% hair loss

2. 25 to ≤50% hair loss

3. 50 to ≤75% hair loss

4. 75% or more hair loss
